# Supplementary material for: Children and adolescents: Respiratory infection and long-term effects longitudinal study (CARE Study): Study protocol
Source: PLoS One. 2026 Feb 5;21(2):e0341566. doi: 10.1371/journal.pone.0341566 (PMC12875511; doi:10.1371/journal.pone.0341566)
Supplement: S1 Appendix — (DOCX) [file pone.0341566.s001.docx]

**Post COVID-19 Condition (PCC) definition***

1. Confirmed SARS-CoV-2 infection
2. Experiencing symptoms lasting at least 2 months, initially occurring within 3 months of acute COVID-19.
3. Symptoms generally impact everyday functioning, such as changes in eating habits, physical activity, behaviour, academic performance, and social functions, as assessed by survey questions and PedsQL scores.
4. Symptoms may be new onset following initial recovery from an acute COVID-19 episode or persist from the initial illness. They may also fluctuate or relapse over time.
5. Follow-up may reveal additional diagnoses, but this does not exclude the diagnosis of post-COVID-19 condition.

**Post-Acute Sequelae (PAS) following influenza infection***

1. Confirmed influenza infection
2. Experiencing symptoms lasting at least 2 months, initially occurring within 3 months of acute influenza.
3. Symptoms generally impact everyday functioning, such as changes in eating habits, physical activity, behaviour, academic performance, and social functions, as assessed by survey questions and PedsQL scores.
4. Symptoms may be new onset following initial recovery from an acute influenza episode or persist from the initial illness. They may also fluctuate or relapse over time.
5. Follow-up may reveal additional diagnoses, but this does not exclude the diagnosis of PAS condition following influenza.

*Adapted from the World Health Organization. A clinical case definition for post-COVID-19 condition in children and adolescents by expert consensus, <https://www.who.int/publications/i/item/WHO-2019-nCoV-Post-COVID-19-condition-CA-Clinical-case-definition-2023-1>.
