# Supplementary material for: Children and adolescents: Respiratory infection and long-term effects longitudinal study (CARE Study): Study protocol
Source: PLoS One. 2026 Feb 5;21(2):e0341566. doi: 10.1371/journal.pone.0341566 (PMC12875511; doi:10.1371/journal.pone.0341566)
Supplement: S3 Appendix — (PDF) [file pone.0341566.s003.pdf]

PROTOCOL

A longitudinal study of post-viral sequelae of common respiratory infections in children and adolescents

Protocol Number:
Version: 1.7
Date: 17/10/2025

Author/s:
Dr Mark McMillan
Dr Nan Vasilunas
Dr Tom Sullivan
Dr Tess Edmond
Dr Ailish Battersby
Dr Philip Britton
Dr Brendan McMullen
Professor Jon Jureidini
Ms Rebecca Beazley
Dr Jeevaki Kuruppu
Professor Helen Marshall

Sponsor:
Women's and Children's Hospital Foundation

Statement of Compliance

This document is a protocol for a research project. This study will be conducted in compliance with all stipulations of this protocol, the conditions of the ethics committee approval, the NHMRC National Statement on ethical Conduct in Human Research (2007) and the Note for Guidance on Good Clinical Practice (CPMP/ICH-135/95).

Table of Contents

Table of Contents 2

1. **Glossary of Abbreviations & Terms** 6
2. **Study Sites** 7
    a. Investigators 7
3. **Introduction/Background Information** 10
    a. Lay Summary 10
    b. Introduction 10
    c. Background information/Literature Review 11
4. **Study Objectives** 15
    a. Hypothesis 15
    b. Study Aims 15
    c. Outcome Measures 16
5. **Study Design** 17
    a. Study Type & Design & Schedule 17
    b. Standard Care and Additional to Standard Care Procedures 17
c. Study methodology 17
d. consumer engagement 24
6. **Study Population** 25
    a. Inclusion Criteria 25
    b. Exclusion Criteria 25
    Participant Recruitment 25
    c. Recruitment Procedure 25
    d. Consent 25
7. **Participant Safety and Withdrawal** 26
    a. Risk Management and Safety 26
    b. Handling of Withdrawals 26
    c. Replacements 27
    d. Distress protocol 27

8. Refer to Appendix A.**Statistical Methods** 27
a. Sample Size Estimation & Justification 27
b. Power Calculations 27
c. Statistical Methods To Be Undertaken 28
9. Storage of Blood and Tissue Samples 28
10. Data Security & Handling 29
a. Details of where records will be kept & How long they will be stored 29
b. Confidentiality and Security 29
c. Ancillary data 29
Appendix A Distress Protocol 30
Appendix B Follow-up of ongoing symptoms process 31
References 32

STUDY SYNOPSIS

(please provide a brief information)

Title:	A longitudinal study of post-viral sequelae following common respiratory infections in children and adolescents
Short Title:	Children and Adolescents: Respiratory Infection and Long-term Effects (CARE Study)
Design:	Longitudinal cohort study
Study Centres:	Women's and Children's Hospital (SA Health notifications)
Hospital:	Women's and Children's Hospital
Study Question:	What is the incidence, duration, and type of post-acute sequelae (PAS) in children and adolescents post-COVID-19 infection?
Study Objectives:	Inform clinical management for children and adolescents, as well as COVID-19 vaccine recommendations, and guide the introduction of interventional studies in South Australian children.
Primary Objectives:	Describe the cumulative incidence, duration, and type of post-acute sequelae following COVID-19 infection (PASC) in children and adolescents at 3 months and the proportion with ongoing symptoms at 6 and 12 months post-COVID-19 infection.
Secondary Objectives	1. Describe the cumulative incidence of influenza infections in children and adolescents at 3 months, and the proportion with ongoing symptoms at 6 and 12 months post-infection.<br>2. Estimate the relative risk of developing PCC following COVID-19 compared to PAS following influenza at 3 months post-infection.<br>3. Establish a model for predicting the development of PCC in children and adolescents with COVID-19.<br>4. Estimate the difference in the quality of life of children and adolescents (aged 2-18) at 3, 6, and 12 months post-infection in the following groups:<br>o PCC compared to those without PCC in COVID-19-positive cases.<br>o PAS compared to those without PAS in influenza-positive cases.<br>o PCC compared to those with PAS<br>5. Estimate the difference in fatigue of children and adolescents (aged 2-18) at 3, 6, and 12 months post-infection in the following groups:<br>o PCC compared to those without PCC in COVID-19-positive cases.<br>o PAS compared to those without PAS in influenza-positive cases.<br>o PCC compared to those with PAS<br>6. Estimate the difference in school absenteeism at 3, 6, and 12 months following infections in the following groups:<br>o PCC compared to those without PCC in COVID-19-positive cases.<br>o PAS compared to those without PAS in influenza-positive cases.<br>o PCC compared to those with PAS

	7. Estimate the difference in influenza vaccine uptake in children and adolescents with PAS following influenza compared to vaccination uptake in those without PAS following influenza.<br>8. Estimate the cumulative incidence of PCC in those with COVID-19 re-infections compared to those with first infection at 3 months, and the proportion with ongoing symptoms at 6 and 12 months post-infection.<br>9. Describe the duration and type of PCC in children and adolescents with COVID-19 infection.<br>10. Describe the duration and clinical features of PAS in children and adolescents following influenza infection.
Inclusion Criteria:	Children and adolescents aged 0 to 18 who have tested SARS-CoV-2 positive (PCR or Rapid Antigen Tests) or influenza (PCR or Rapid Antigen Tests) in the previous 2 months are eligible for enrollment.
Exclusion Criteria:	• Inability to give informed consent<br>• No mobile phone number<br>• Children and adolescents who are under the care of the statewide WCHN Palliative care service
Number of Planned Subjects:	Assuming 15% participation, based on response rates from similar studies, there would be approximately 1,222 COVID-19 cases, with 2,800 non-COVID-19 cases from SA Health notification over the 24 months of the study.
Investigational product:	N/A
Safety considerations:	N/A
Statistical Methods:	Analyses will follow a pre-specified statistical analysis plan. The cumulative incidence of PCC following COVID-19 (primary objective) and PAS following influenza will be estimated at 3 months post-infection, with 95% confidence intervals. The proportion of individuals with ongoing symptoms will be reported descriptively (n, %) at 6 and 12 months. The relative risk of developing PCC following COVID-19 versus developing PAS following influenza (objective 2) will be estimated using a log-binomial model. A multivariable logistic model will be developed for predicting PCC at 3 months (objective 3), incorporating pre-specified predictors such as age (treated as continuous), sex, pre-existing conditions, and socioeconomic status. Model performance will be evaluated using the area under the receiver operating characteristic curve, with internal validation by bootstrap resampling in line with TRIPOD recommendations.[1] The relatively small expected number of PCC events may limit the number of predictors that can be incorporated into the final model and hence its predictive accuracy. Linear and negative binomial mixed-effects models will assess differences between comparator groups over time in continuous (PedsQL quality of life and fatigue scores, objectives 4 and 5) and count (days absent, objective 6) outcomes, respectively. Log binomial models will compare the risk of developing PAS according to influenza vaccine uptake (objective 7) and the risk of PCC by COVID-19 reinfection (objective 8). The duration and clinical features of PCC and PAS will be described using summary statistics, with summaries at 3, 6 and 12 months. Descriptive analyses will be based on complete case data.
Subgroups:	N/A

1. GLOSSARY OF ABBREVIATIONS & TERMS

Abbreviation	Description (using lay language)
COVID-19	SARS-CoV-2 virus
AIR	Australian Immunisation Register
CALHN	Central Adelaide Local Health Network
CNS	Central nervous system
DALY	Disability-adjusted life years
EMR	Electronic medical records
GBS	Guillain-Barre syndrome
IPD	Individual patient data
ISARIC	International Severe Acute Respiratory and Emerging Infection Consortium
LDN	Low-dose naltrexone
MAPS	Malmo POTS Score
MIS-C	Multisystem inflammatory syndrome in children
PAEDS	Paediatric Active Enhanced Disease Surveillance
PAS	Post-acute sequelae
PASC	Post-acute sequelae from SARS-CoV-2 virus
PCC	Post COVID-19 condition
PCR	Polymerase chain reaction
PedsQL	Pediatric Quality of Life Inventory
PIMS-TS	Paediatric inflammatory, multisystem syndrome temporally associated with SARS-CoV-2
POTS	Postural orthostatic tachycardia syndrome
SCHN	Sydney Children's Hospitals Network
TRIPOD	Transparent reporting of a multivariable prediction model for individual prognosis or diagnosis
UK	United Kingdom
VIRTU	Vaccinology and Immunology Research Trials Unit
WCHN	Women's and Children's Health Network
WHO	World Health Organization
YLD	Years lived with disability
YLL	Years of life lost

2. STUDY SITES

a. INVESTIGATORS

Principal Investigator		Role in Study
Name:	Dr Mark McMillan	Study design, protocol development, HREC approvals, study management, milestone reporting, adverse event management and reporting, data collection, management, analysis and interpretation, manuscript preparation and dissemination.
Position:	Post Post-Doctoral Research Associate	
Department:	University Department of Paediatrics	
Organisation:	Women's and Children's Hospital Network	
E-mail:	mark.mcmillan@adelaide.edu.au	
Telephone:		

Investigator		Role in Study
Name:	Dr Nan Vasilunas	Study design, protocol development, data interpretation, and manuscript preparation.
Position:	Paediatric Infectious Diseases Consultant	
Department:	Microbiology and Infectious Disease	
Organisation:	Women's and Children's Hospital Network	
E-mail:	nan.vasilunas@sa.gov.au	
Telephone:		

Investigator		Role in Study
Name:	Dr Tom Sullivan	Study design, protocol development, data interpretation, and manuscript preparation.
Position:	NHMRC Emerging Leadership Fellow SAHMRI Women and Kids	
Department:	South Australian Health & Medical Research Institute (SAHMRI) Women and Kids	
Organisation:	SAHMRI	
E-mail:	thomas.sullivan@sahmri.com	
Telephone:		

Investigator		Role in Study
Name:	Dr Tess Edmond	Study design, protocol development, data interpretation, and manuscript preparation.
Position:	General Paediatrician	
Department:	University Department of Paediatrics	
Organisation:	Women's and Children's Hospital Network	
E-mail:	tess.edmond@adelaide.edu.au	
Telephone:		

Investigator		Role in Study
Name:	Dr Ailish Battersby	Study design, protocol development, data interpretation, and manuscript preparation.
Position:	General Paediatrician	
Department:	Department of General Medicine	
Organisation:	Women's and Children's Hospital Network	
E-mail:	ailish.battersby@sa.gov.au	
Telephone:		

Investigator		Role in Study
Name:	Dr Philip Britton	Study design, protocol development, data interpretation, and manuscript preparation.
Position:	Staff Specialist & Conjoint Associate Professor	
Department:	Department of Infectious Diseases and Microbiology & Child and Adolescent Health	
Organisation:	Children's Hospital Westmead & Sydney Medical School, University of Sydney	
E-mail:	philip.britton@health.nsw.gov.au	
Telephone:		

Investigator		Role in Study
Name:	Dr Brendan McMullan	Study design, protocol development, data interpretation, and manuscript preparation.
Position:	Paediatric Infectious Diseases Specialist and Microbiologist	
Department:	Department of Immunology and Infectious Diseases	
Organisation:	Sydney Children's Hospital, Randwick	
E-mail:	brendan.mcmullan@health.nsw.gov.au	
Telephone:		

Investigator		Role in Study
Name:	Professor Jon Jureidini	Study design, protocol development, data interpretation, and manuscript preparation.
Position:	Child Psychiatrist	
Department:	Critical and Ethical Mental Health (CEMH) research group within the Robinson Research Institute	
Organisation:	University of Adelaide	
E-mail:	jon.jureidini@adelaide.edu.au	
Telephone:		

Investigator		Role in Study
Name:	Professor Helen Marshall	Study design, protocol development, data interpretation, and manuscript preparation.
Position:	Consultant in Vaccinology & Medical Director, VIRTU	
Department:	University Department of Paediatrics	
Organisation:	Women's and Children's Hospital Network	
E-mail:	helen.marshall@adelaide.edu.au	
Telephone:		

Investigator		Role in Study
Name:	Rebecca Beazley	Study design, protocol development, data interpretation, and manuscript preparation.
Position:	Manager, Disease Surveillance and Investigation Section	
Department:	Communicable Disease Control Branch	
Organisation:	SA Health	
E-mail:	Rebecca.Beazley@sa.gov.au	
Telephone:		

Investigator		Role in Study
Name:	Dr Jeevaki Kuruppu	Participant follow up, data interpretation, protocol revision, and manuscript preparation.
Position:	Senior Research Medical Officer	
Department:	University Department of Paediatrics	
Organisation:	Women's and Children's Hospital Network	
E-mail:		
Telephone:		

3. INTRODUCTION/BACKGROUND INFORMATION

a. LAY SUMMARY

This study investigates ongoing symptoms following viral respiratory infections in children and teenagers, including COVID-19 (long COVID).

While there is substantial data on these viruses' immediate effects, understanding the long-term health implications, especially among children and teenagers, is limited. The existing knowledge is mainly based on international studies. However, the situation in Australia might be different due to factors such as vaccination rates and the prevalence of different COVID-19 variants.

Children with respiratory infections will be invited to participate in the study.

After providing consent, the participants (or their parents) will be asked to complete surveys at the start, then 3, 6, and 12 months after a viral respiratory infection. This will allow the researchers to monitor health issues that continue over time.

The study's primary goal is to discover how common these ongoing health problems are among young people and how they affect their daily lives.

The results of this research are expected to provide important information that may guide decisions about how to care for young people who experience health problems after respiratory infections. The study will also examine how these ongoing health issues affect school and if some children are more at risk of having longer-term problems.

In short, the aim is to get clearer information about the ongoing health issues children and teenagers can experience after respiratory infections to improve the health care they receive.

b. INTRODUCTION

Viruses significantly impact human health worldwide. For instance, the SARS-CoV-2 virus (COVID-19) has caused more than 7 million deaths since its emergence in late 2019.[2] While some viruses, such as herpesvirus, establish lifelong latent infections with potential for reactivations, most lead to transient acute infections. During this acute phase, acute symptoms broadly correlate with the period during which viral replication occurs. However, a substantial body of research indicates that complications and clinical manifestations can persist beyond the acute phase for many viruses, a phenomenon known as post-acute sequelae (PAS). Some PAS symptoms resolve over time, and others can last a lifetime.[3]

Ongoing complications following acute COVID-19 infection, known as post-acute sequelae from COVID-19 (PASC), post COVID-19 condition (PCC), or 'long COVID', were initially believed to be rare in school-aged children. From this point forward this complication will be referred to as post COVID-19 condition (PCC). However, recent international studies have described a notably higher burden lasting up to 12 months after the acute illness. A pooled estimate from 27 international cohort studies estimated that 16.2% (95% CI 8.5% to 28.6%) of children and adolescents had persistent symptoms at least 3 months after infection. Symptoms most commonly included sore throat, persistent fever, sleep disturbances, fatigue, and muscle weakness.[4] However, there are significant limitations in the current literature due to the non-specific nature of symptoms, different definitions used to classify PCC, and challenges in comparing cases to control groups.[4]

Most evidence for PCC is from international studies, and Australia's situation might differ. High vaccination rates coupled with Omicron variant predominance amongst total primary infections may contribute to a different incidence of PCC compared to that in other nations. Even with a possible low incidence of PCC in Australian children and adolescents, the large number of infections and repeat infections may still result in a substantial PCC burden. A significant gap persists in understanding the burden of PCC, and there is currently no comprehensive strategy for PCC data collection in Australia, which is crucial for managing the condition.

This research study is designed as a longitudinal cohort study. Children and adolescents (0-18 years) with positive SARS-CoV-2 and influenza infections will be invited to participate. Parents of children and adolescents will be asked to complete surveys at baseline, 3, 6, and 12 months to identify post-acute sequelae. The study aims to determine the incidence, disease spectrum, burden, and duration of PCC in children and adolescents at 3, 6, and 12 months after infection in South Australia. The study will ultimately help inform clinical management for children and adolescents, possibly COVID-19 vaccine guidelines, and facilitate the roll-out of interventional studies.

c. Background information/Literature Review

Two and a half years into the pandemic, it is evident that ongoing complications persist across multiple body systems, in the months following SARS-CoV-2 infection, known as post-COVID-19 condition resulting from COVID-19 infections (PCC), or long COVID.[5] Considerable uncertainty remains about the incidence, diagnosis, treatment, and prevention of PCC. The lack of evidence is even starker in children and adolescents, who, in some respects, have been a secondary consideration in the pandemic due to the highest burden of disease occurring in older adults.

COVID-19 and other acute respiratory infections are associated with a range of symptoms more than four weeks after the acute infection.[6] PCC has primarily been described in adults with ongoing symptoms following COVID-19. Multiple systems can be affected, including sensory, neurologic, and cardiorespiratory.[7] The clinical course of COVID-19 infection differs for children and adolescents compared to adults. For each confirmed COVID-19 infection in children under 16 years of age in Australia, 1.38% (95% CI: 1.17% to 1.59%) were admitted to hospitals for medical care directly related to the SARS-CoV-2 infection from June to November 2021 (Delta), with 0.09% of these were admitted to intensive care.[7] A much larger burden of disease is seen in older adults (Figure 1).[8]

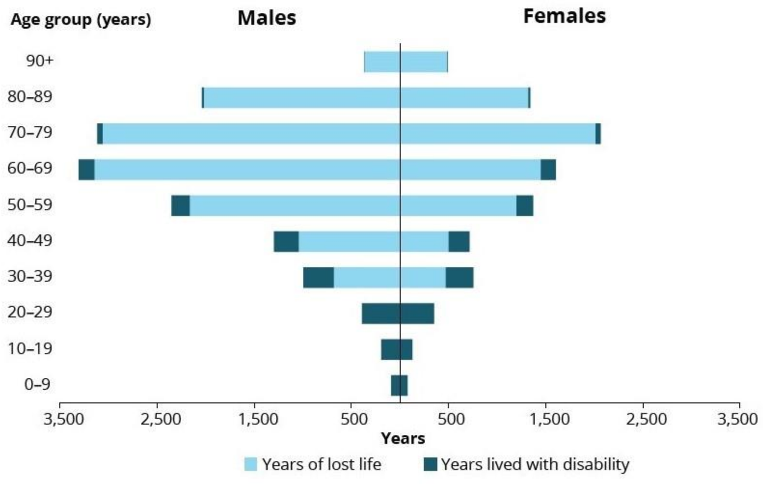

Source: AIHW analyses as detailed in AIHW 2021b.

Figure 1. Total burden (DALYs) for COVID-19 in Australia, disaggregated into fatal (YLL) and non-fatal (YLD) burden by sex, 2021.[7]

Another difference between children and adults is the occurrence of the post infectious inflammatory condition known as multisystem inflammatory syndrome in children (MIS-C) or paediatric inflammatory, multisystem syndrome temporally associated with SARS-CoV-2 (PIMS-TS). The rare hyperinflammatory state (1 in every 2,500) is predominantly seen in children, although adult cases have been reported.[9,10] MIS-C manifests approximately 2 to 6 weeks post the initial SARS-CoV-2 infection, presenting symptoms like fevers, rashes, gastrointestinal issues, and elevated inflammatory markers. It can induce inflammation in the heart, lungs, kidneys, brain, skin, and eyes. Since 2020, 18 cases have been reported at the WCHN.[10]

Follow-up studies on MIS-C indicate that the majority of patients recover fully, including improvement of cardiac dysfunction. Some have suggested that PCC in children is a subclinical presentation of MIS-C.[11] However, elevated markers of inflammation, a central feature of MIS-C, are uncommon in long COVID. Furthermore, there is limited evidence that immunomodulatory treatment, which is very effective in managing MIS-C, has a role in treating long COVID. There are some studies on low-dose naltrexone (LDN) that suggest some patients experience fewer number of symptoms, and improved clinical symptoms, however randomised placebo controlled trials are required.[12,13] LDN can act as an immunomodulator in multiple autoimmune diseases and malignant tumors.[14] Given the distinct differences between children and adults with COVID-19 infection, paediatric-specific research on PCC is essential.[11]

In September 2022, a panel of 27 experts convened to establish a clinical case definition for the post-COVID-19 condition in children and adolescents, informed by the scientific data available at that time. This definition is expected to undergo revisions as further high-quality evidence emerges and our collective understanding of the condition deepens.[15] The World Health Organization (WHO) defines PCC, as:

- Occurring in individuals with a history of confirmed or probable SARS-CoV-2 infection
- Symptoms last at least 2 months, initially occurring within 3 months of acute COVID-19
- Symptoms generally impact everyday functioning, such as changes in eating habits, physical activity, behaviour, academic performance, social functions (interactions with friends, peers, and family) and developmental milestones.
- Symptoms may be new onset following initial recovery from an acute COVID-19 episode or persist from the initial illness.
- They may also fluctuate or relapse over time.
- Workup may reveal additional diagnoses, but this does not exclude the diagnosis of post-COVID-19 condition.
- More frequently reported symptoms in children compared with controls are fatigue, altered smell/anosmia and anxiety. Other symptoms have also been reported.
- This definition can be applied to children of all ages, with age-specific symptoms and impact on everyday function considered.

There is no such definition for post-acute sequelae of influenza. However, it is noted that adults can experience central nervous system (CNS) syndromes, such as Guillain-Barre Syndrome (GBS), and a worsening of underlying conditions like ischemic heart disease and cerebrovascular disease following influenza infection. These health issues can manifest weeks to months after recovery.[16]

A recent cross-sectional study that conducted a one-off phone survey involving Queensland adults revealed minimal differences in post-acute sequelae between COVID-19 and influenza patients 12 weeks after an Omicron outbreak. However, it remains unclear if and how recently the individuals in the influenza group might have had COVID-19 infections.[17] Additional studies have made comparisons between post-acute sequelae following COVID-19 and influenza. A 6-month retrospective cohort study utilising electronic medical records (EMR) of 236,379 COVID-19 patients and 105,579 diagnosed with influenza identified higher rates of anxiety and mood disorders, insomnia, and dementia post-COVID-19 than post-influenza.[18] Another study, which analysed EMRs from American veterans (88% male), reported increased rates of sequelae in multiple body systems following COVID-19 compared to influenza, including neurological and neurocognitive disorders, mental health disorders, cardiovascular disorders, gastrointestinal disorders, coagulation disorders, and other disorders including malaise and fatigue.[19]

Four systematic reviews have been performed during different pandemic periods,[20-23] with the two most recent ending their search in December 2022.[20,23] The systematic review with the most comprehensive search strategy and relevant selection criteria included 27 cohort studies (19 prospective cohorts, 8 retrospective cohorts, and 4 cross-sectional studies), with approximately 15,000 paediatric participants aged between 0 and 19 years of age.[20] The majority of studies included were conducted in Europe (n=13), with the remaining from Iran (n=2), Australia (n=1), China (n=1), the United States (n=1), and one from multiple countries. The systematic review reported that 16.2% (95% CI 8.5% to 28.6%) of children and adolescents experienced one or more persistent symptoms at least 3 months post-infection (follow-up duration 3 to 13 months). The five most prevalent long-term clinical manifestations after COVID-19 in this demographic were sore throat, persistent fever, sleep disturbance, fatigue, and muscle weakness.[20]

Concerningly, in the latest findings from the United Kingdom's (UK) CLoCk Study, which carried out a longitudinal cohort study on 11-17 year-olds with both negative and positive COVID-19 tests, young people with COVID-19 re-infection had more symptoms and a higher prevalence of symptoms than those with one infection.[5] Another survey led by the Office for National Statistics in the UK of over 500,000 participants from the UK has shown no reduction of risk of developing long COVID in <16 year-olds for those experiencing a second infection, compared to the first infection.[24] Evidence of risk factors associated with PCC in children is limited, with adolescents, females, and those with more severe disease (48 hours or more in hospital) potentially being at higher risk.[23]

Only two studies are available from Australia, with one study of 171 children from Melbourne[25] and another conducted in Sydney, which is currently in preprint.[26] The first was conducted at the Royal Children's Hospital in Melbourne for approximately 12 months starting in March 2020. A standardised clinic proforma collected information on acute and post-acute COVID-19 symptoms. At 3 to 6 months, follow-up data were available for 151/171 (88%) who enrolled. The most common post-acute COVID-19 symptoms were mild post-viral cough (6/151 [4%]), fatigue (3/151 [2%]) and post-viral cough and fatigue (1/151 [1%]).[25]

The second study, conducted at the Sydney Children's Hospitals Network (SCHN), involved contacting 9,765 eligible children (aged 0-18 years) and their families during the 2021 Delta outbreak.[26] Out of these, 1,731 responded. Participants were given a 15-minute online RedCap survey to complete. The results indicated that 6.1% (106/1,731) experienced persistent COVID-19-related symptoms at 3 months. Furthermore, 3.0% (52/1,730) could not resume their usual activities, and 1.7% (30/1,730) required extra assistance to recover from

COVID-19.[26] From the responses, 203 met the 'criteria for concern'. Of these, 83% (169 individuals) received follow-up from a clinician. Based on further examination, the authors determined that 63 children exhibited signs of a post-COVID condition. Most of these children presented with upper or lower respiratory tract issues, multi-organ persistent symptoms, or fatigue. Of these, 21 children (representing 1.2% of the responder cohort with a 95% CI of 0.8-1.8) had symptoms comparable with the UK consensus definition.[26]

By the end of 2022, approximately 450,000 children under 9 and 575,000 adolescents aged 10-19 in Australia had confirmed COVID-19 infections.[27] Using 1.2% to 16% as a guide, this translates to around 12,300 to 166,000 Australian children experiencing post-acute sequelae lasting 3 months or longer, which does not account for repeat infections. However, in Australia, the combination of high vaccination rates and the Omicron variant predominance amongst total primary infections suggest that the incidence of long COVID might differ from that in other countries. Even with a lower proportion of PCC in children and adolescents, the absolute number of infections indicates a need for a more coordinated and informed approach to managing PCC in Australian children and adolescents. The authors of the SCHN study highlight the need to develop pathways to support screening of children for long COVID and provide better guidance for primary care services.[26] They also point out that there is currently no comprehensive approach to collect data on PCC across the lifespan, which is required to inform management of the condition and implementation of interventional studies.[26] While there is currently no known effective treatment, COVID-19 vaccination has demonstrated some protection in adults [28] and children aged 5-17, (effectiveness of 41.7% (15.0 - 60.0) against diagnosed long COVID), although it is less effective in younger age groups, and not effective at 18 months since the last vaccination.[29] Many pharmaceutical candidates are undergoing trials to establish if they are an effective therapeutic intervention.[30]

The Department of Health and Aged Care's Post-Acute Sequelae of COVID-19 Research Plan, which focuses on effective management, not primary prevention, has highlighted knowledge gaps in 1) understanding the short to long-term effects of long COVID on individuals, communities, and health systems, as well as 2) knowledge gaps in understanding critical factors influencing long COVID prognosis.[31] Multiple global working and expert groups have published priorities for research in long COVID and repeat infection.[32-35] In South Australia, adults can access coordinated care for post-acute sequelae at the Long COVID Assessment Clinic, albeit with a substantial waiting list.[36] However, there is no parallel service for children. Understanding how children, young adults, and their families navigate the health system for timely, evidence-based support is limited. Additionally, while factors such as age, gender, pre-existing conditions, and severity of initial infections influence the risk of post-acute sequelae,[23] the determinants affecting the incidence and severity of long COVID in Australian children and adolescents remain unclear.

The research priorities outlined by the WHO expert working group on PCC in children and adolescents focus on several key aspects: assessing the effect of symptoms on daily activities across various age groups, determining if certain symptoms tend to occur in clusters, comprehending the persistence and evolution of symptoms over time, and evaluating the impact of variants of concern and the effect of re-infection.[15]

This longitudinal case-cohort study aims to identify the incidence and predictors of PCC in Australian children and adolescents. We plan to recruit children and adolescents with respiratory infections from the Women's and Children's Hospital, then expand across a broader SA-wide cohort. After obtaining informed consent, we will send questionnaires to participants at baseline, 3, 6, and 12 months. These questionnaires will assess various

factors, including incidence, vaccination history, risk factors, severity, healthcare utilisation, quality of life, and fatigue in participants under the age of 18.

Existing literature recommends that longitudinal cohort studies include control groups and ensure the recording of symptoms at consistent follow-up intervals. It is also essential to consider pre-existing medical conditions.[4] Our study design addresses the current shortfall in rigorously structured research.

Our research aims to inform clinical management for children and adolescents, as well as COVID-19 vaccine recommendations, and guide the introduction of interventional studies in South Australian children.

4. STUDY OBJECTIVES

a. HYPOTHESIS

That the absolute number of infections in South Australia indicates a need for a more coordinated and informed approach to managing PCC in children and adolescents.

b. STUDY AIMS

Primary objectives

1. Describe the cumulative incidence of PCC in children and adolescents at 3 months and the proportion with ongoing symptoms at 6 and 12 months post-COVID-19 infection

Secondary objectives

1. Describe the cumulative incidence of influenza infections in children and adolescents at 3 months, and the proportion with ongoing symptoms at 6 and 12 months post-infection.
2. Estimate the relative risk of developing PCC following COVID-19 compared to PAS following influenza at 3 months post-infection.
3. Establish a model for predicting the development of PCC in children and adolescents with COVID-19.
4. Estimate the difference in the quality of life of children and adolescents (aged 2-18) at 3, 6, and 12 months post-infection in the following groups:
    - PCC compared to those without PCC in COVID-19-positive cases.
    - PAS compared to those without PAS in influenza-positive cases.
    - PCC compared to those with PAS
5. Estimate the difference in fatigue of children and adolescents (aged 2-18) at 3, 6, and 12 months post-infection in the following groups:
    - PCC compared to those without PCC in COVID-19-positive cases.
    - PAS compared to those without PAS in influenza-positive cases.
    - PCC compared to those with PAS
6. Estimate the difference in school absenteeism at 3, 6, and 12 months following infections in the following groups:

- PCC compared to those without PCC in COVID-19-positive cases.
- PAS compared to those without PAS in influenza-positive cases.
- PCC compared to those with PAS

7. Estimate the difference in influenza vaccine uptake in children and adolescents with PAS following influenza compared to vaccination uptake in those without PAS following influenza.
8. Estimate the cumulative incidence of PCC in those with COVID-19 re-infections compared to those with first infection at 3 months, and the proportion with ongoing symptoms at 6 and 12 months post-infection.
9. Describe the duration and clinical features of PCC in children and adolescents with COVID-19 infection.
10. Describe the duration and clinical features of PAS in children and adolescents following influenza infection.

c. Outcome Measures

Primary

1. Cumulative incidence of PCC at 3 months in children who tested COVID-19 positive (PCR, or RAT), and the proportion with PCC at 6 and 12 months post-infection will be assessed from survey responses and follow-up.

Secondary

1. Incidence of PAS at 3 months in children following influenza (PCR or RAT) and the proportion with PAS following influenza at 6 and 12 months post-infection from survey responses and follow-up
2. The relative risk comparing the cumulative risk of meeting the case definition for PCC among participants with COVID-19 to the cumulative risk of meeting the case definition for PAS among participants with influenza from survey responses and follow up.
3. A multivariable logistic model will be developed for predicting PCC at 3 months, incorporating pre-specified predictors such as age (treated as continuous), sex, pre-existing conditions, and socioeconomic status.
4. Difference in the quality of life of children and adolescents (aged 2-18) using the Pediatric Quality of Life Inventory (PedsQL) short form (scores 0-100) in the following groups:
    - PCC compared to those without PCC in COVID-19-positive cases.
    - PAS compared to those without PAS in influenza-positive cases.
    - PCC compared to those with PAS
5. Difference in fatigue of children and adolescents (aged 2-18) at 3, 6, and 12 months using the PedsQL Multidimensional Fatigue Scale (scores 0-100), in the following groups:
    - PCC compared to those without PCC in COVID-19-positive cases.

- PAS compared to those without PAS in influenza-positive cases.

  PCC compared to those with PAS

6. Difference in school absenteeism in the previous month at 3, 6, and 12 months from the self-reported survey, in the following groups:
   - PCC compared to those without PCC in COVID-19-positive cases.
   - PAS compared to those without PAS in influenza-positive cases.
   - PCC compared to those with PAS
7. The difference in vaccine uptake (confirmed using the Australian Immunisation Register) in those with PAS compared to those without PAS in influenza-positive cases.
8. Proportion of participants meeting the case definition for PCC at 3 months following COVID-19 infection, by infection history (first infection vs. reinfection).
9. Description of the duration and type of PCC in those with COVID-19 infections. Derived from 3, 6, and 12 month survey responses.
10. Description of the duration and clinical features of PAS in children and adolescents following influenza infection. Derived from 3, 6, and 12 month survey responses.

5. STUDY DESIGN

a. STUDY TYPE & DESIGN & SCHEDULE

This study is designed as a longitudinal cohort study. The study will recruit participants for 24 months from study commencement, and each participant will be enrolled for 12 months.

b. STANDARD CARE AND ADDITIONAL TO STANDARD CARE PROCEDURES

Standard care for individuals under 18 years of age who experience ongoing sequelae from a COVID-19 infection or non-COVID respiratory infections primarily involves assessment through general practice or hospital outpatient services. All children who report severe persistent symptoms who have not yet undergone a medical assessment with functional impairments (WHO definition)[15] will be contacted for a Nurse-led or WCHN employed Paediatrician via a telehealth call to further clinically assess cases and determine if a referral to their General Practitioner is required. Refer to Appendix B for details regarding Nurse-led or WCHN employed Paediatrician follow up process. South Australia does not have a specialised long COVID clinic or management protocol for children and adolescents.

c. STUDY METHODOLOGY

The Vaccinology and Immunology Research Trials Unit conducts surveillance of SARS-CoV-2, and influenza infections at the Women's and Children's Hospital as part of the Paediatric Active Enhanced Disease Surveillance (PAEDS) network.[37]

Similar to current methods used by the PAEDS network, for phase 1 of the study WCHN pathology results will be searched each fortnight by a WCHN employed level 2 Registered Nurse, and SARS-CoV-2 test-positive and influenza test-positive children and adolescents (aged 0-18 years) will be invited to participate (Figure 1).

For phase 2 of the study, SARS-CoV-2, and influenza infections will be recruited from SA Health notifications of PCR or Rapid Antigen Tests (RAT), to ensure the results are generalisable to the South Australian populations and include children and adolescents with potentially milder acute illness. Once recruitment commences through SA Health notifications, this will replace invitations sent from WCHN to avoid duplication of invitations.

COVID-19 and influenza cases parents will be sent an SMS invitation, and if they consent, agree to 1) completing a baseline survey, then surveys at 3, 6, and 12 months, 2) for the research team to review EMR if they present to a SA Health hospital (WCHN, FMC, and LMH), and permission for the study team to access the Australian Immunisation Register to check COVID-19, and influenza immunisation status.

To reimburse participants for their time and encourage the completion of surveys, a $10 online supermarket voucher will be provided upon completion of the initial, then 3, 6, and 12-month surveys for their time ($40 in total). If parents deem that their 12-18-year-olds are mature enough to understand the relevant information and to give consent, assent will also be collected if they wish to complete the surveys themselves. If the 12-18 year old does not agree to participate from the study, they will be withdrawn from the study.

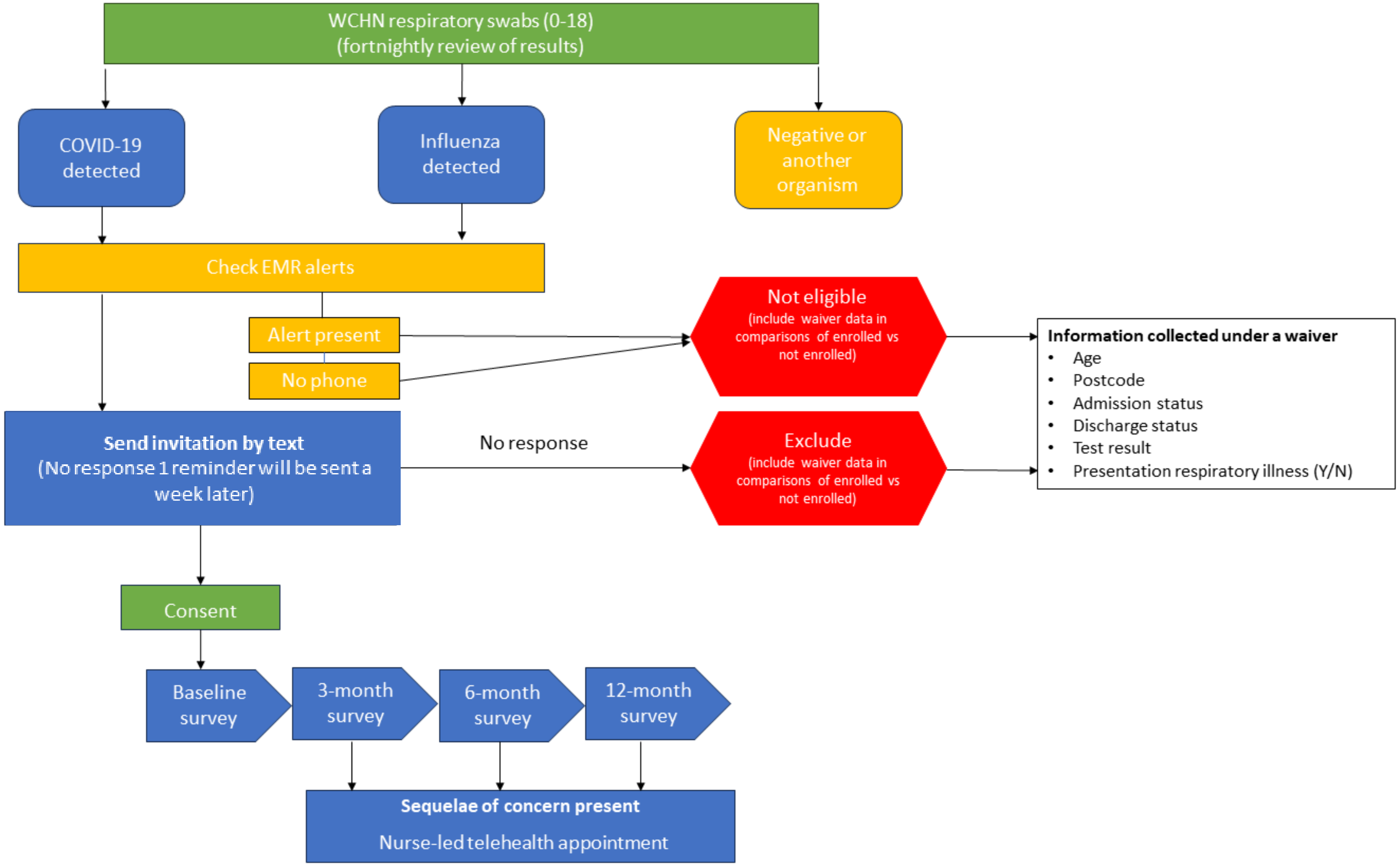

Figure 2: Recruitment flow chart for WCHN (Phase 1 of recruitment)

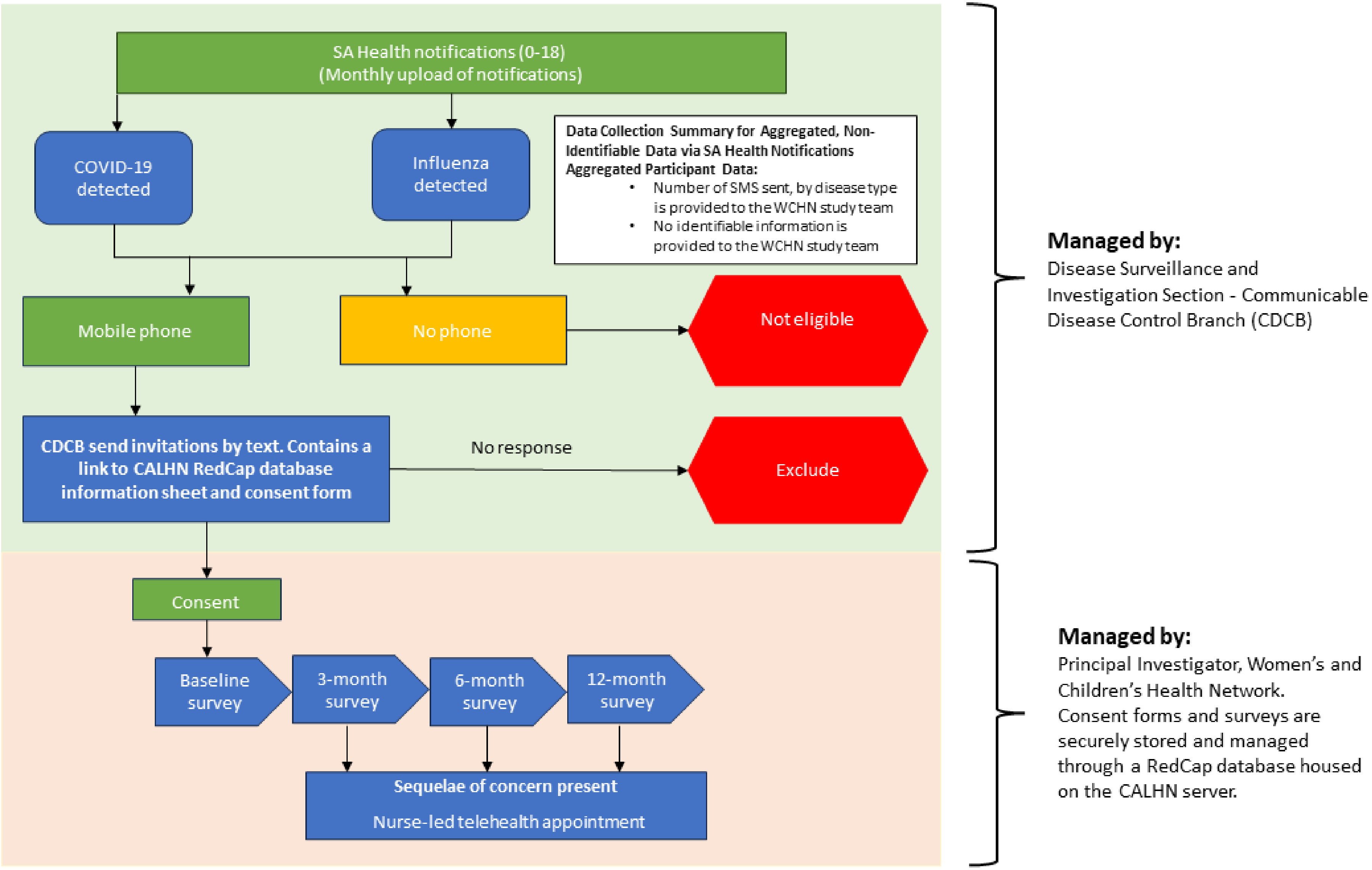

Figure 3: Recruitment flow chart for SA Health notifications <u>(Phase 2 of recruitment)</u>

Data collection (Phase 1 of WCHN recruitment)

Following a waiver of consent, basic baseline information of all COVID-19 and influenza cases presenting to a WCHN site will be collected by a WCHN employed level 2 Registered Nurse from EMR to provide baseline denominator data for those not enrolled in the study. Their name and phone number are also required to send the initial invitation. This information will include:

- Name
- Date of birth
- Phone number
- Swab date
- Postcode
- Type of positive test
- Admitted to hospital Y/N
- Presentation for respiratory illness (Y/N)
- Discharge status

Data collection (Phase 2 of Communicable Disease Control Branch [CDCB] recruitment)

The Disease Surveillance and Investigation Section at the CDCB routinely collects notification data for influenza and COVID-19. No additional data will be collected, and notification data will not be shared with the WCHN study team. CDCB will only provide the WCHN study team with the aggregated number of SMS invitations sent for influenza and COVID-19.

The SMS invitation will contain a link to the participant information sheet and consent form, which are securely stored on a RedCap database housed on the CALHN server and managed by the WCHN study PI. The WCHN study team will only have access to information from individuals who have provided consent.

Data from consented participants

Following consent, survey data will be collected from those enrolled using an online RedCap baseline questionnaire to be completed at recruitment, followed by questionnaires at 3, 6, and 12 months post-infection. The WCHN study team will validate the positive test results of consented participants with CDCB. Questionnaires will be available for parents/carers or participants to answer where applicable. Quality of life questions will be available in in two of the fasting-growing non-English languages used at home in South Australia: Mandarin, and Vietnamese.[38] The survey is based on the International Severe Acute Respiratory and Emerging Infection Consortium (ISARIC) COIVD-19 Paediatric follow-up protocol and questionnaire.[39] If there is no response from a survey after one week, 2 reminders will be sent a week apart. Data will be collected using a secure password-protected web application housed on a SA Health data server (RedCap).

Data to be collected includes:

Baseline data (1 month after swab result or hospital discharge)

- Name of person filling in the questionnaire and relationship with the case.
- Sex/gender

- Ethnicity
- Medically diagnosed conditions prior to respiratory infection
- Symptoms of acute COVID-19 infection (first 14 days)
- History of confirmed SARS-CoV-2 tests

Collected by study staff:

- Vaccination history (confirmed on the Australian Immunisation Register)
- If hospitalised (from EMR):
    - Length of stay
    - Number of viral infections
    - Treatment, e.g., antivirals, antibodies
    - Pneumonia Y/N
    - ICU/HDU admission Y/N
    - Respiratory support Y/N

3, 6, and 12 months (Online surveys)

- Name of person filling in the questionnaire and relationship with the case.
- PCR/RAT tests and results since the last survey
- New conditions diagnosed by GP since the last survey
- Physical or mental health symptoms since the last survey
    - List of common long COVID symptoms, including questions about symptom intensity, based on the ISARIC Global Tier 1 Ongoing COVID-19 Follow-Up Survey symptom list.
    - Malmo POTS Score (MAPS).[40]
    - Medical follow up of these symptoms
- Days absent from school, work, or higher education in the last month
- Conditions that have required follow-up with a GP
- Readmission to hospital
- Pediatric quality of life inventory (PedsQL) short form. The 15 items in the PedsQL comprise four Generic Core Scales (for ages 2 to 18):[41]
    - Physical Functioning
    - Emotional Functioning
    - Social Functioning
    - School Functioning
- The PedsQL Multidimensional Fatigue Scale is composed of 18 items across three dimensions:[42]

  - General fatigue
  - Sleep/rest fatigue
  - Cognitive fatigue

Collected by study staff:

- If hospitalised with respiratory illness (From EMR):
  - Length of stay
  - Number of viral infections
  - Treatment, e.g., antivirals, antibodies
  - Pneumonia Y/N
  - ICU/HDU admission Y/N
  - Respiratory support Y/N

Table 1. Study time points

Information collected	Waiver of consent (Phase 1 WCHN only)	Survey 1 (Baseline)	Survey 2 (3 months)	Survey 3 (6 months)	Survey 4 (12 months)
Name	x				
Date of birth	x				
Postcode	x				
Mobile phone number	x				
Swab date and details	x				
Hospitilisation information<br>• Admission to hospital<br>• Discharge status<br>• Related to viral infection (Y/N)	x				
Consent and assent (if applicable)		x			
Demographic details		x			
History of COVID-19 infection		x	x	x	x
Medical history		x	x	x	x
Symptoms of acute infection		x			
Physical and mental health symptoms		x	x	x	x
Medical follow up of symptoms			x	x	x
Vaccination history (from AIR)		x			
Days absent from school/work		x	x	x	x
GP presentations since the last survey			x	x	x
Information from EMR if hospitalised<br>• Length of stay<br>• Number of viral infections<br>• Treatment e.g., antivirals, antibodies<br>• Pneumonia Y/N<br>• ICU/HDU admission Y/N<br>• Respiratory support Y/N		x	x	x	x
Pediatric quality of life inventory (PedsQL).			x	x	x
PedsQL Multidimensional Fatigue Scale			x	x	x

d. CONSUMER ENGAGEMENT

The study has two consumers with lived experience of caring for a child with ongoing sequelae following COVID-19 infection. The consumers have undergone an onboarding session to become WCHN Consumer Advocates. They have agreed to the role requirements and have signed necessary agreements, including codes of conduct and confidentiality.

The consumers will meet regularly with the Principal Investigator, who will report their feedback to the investigator team. They will also meet with the investigator team at regular intervals. The consumer advocates will be involved in reviewing all study material, including the protocol, PI sheet, consent form, questionnaires, and communications with potential participants and participants. The consumers will also be involved in interpreting results, planning communication strategies, and disseminating the results. The consumers are reimbursed for their time according to the standard consumer sitting fee of the WCHN consumer advocates.

6. Study Population

a. Inclusion Criteria

1) Children and adolescents aged 0 to 18 who have tested SARS-CoV-2 positive (PCR or Rapid Antigen Tests) or influenza (PCR or Rapid Antigen Tests) in the previous 3 months are eligible for enrollment.

b. Exclusion Criteria

1) Inability to give informed consent
2) No mobile phone number
3) Children and adolescents who are under the care of the statewide WCHN Palliative care service

Participant Recruitment

c. Recruitment Procedure

Phase 1 WCH: Each fortnight, parents of children aged 0 to 18 will be contacted if their child is identified as having a positive PCR or Rapid Antigen Test (RAT) for COVID-19 or influenza infection at the WCHN. They will be sent one phone text message informing them about the study and inviting them to participate using an online link. The link will take them to the online information sheet and consent for the study.

Before contacting the parents of WCHN hospitalised children, the electronic medical record will be checked to ensure no alerts are present.

Phase 2 SA Health Notifications: SA Health receive notifications of SARS-CoV-2 PCR. They also receive notification of influenza PCR-positive cases. Every month, the Disease Surveillance and Investigation Section team (CDCB) collates these notifications for COVID-19 and influenza. A list of children who have accessed the statewide WCHN palliative care service will be cross-matched with influenza and COVID-19 notifications. Influenza and COVID-19 notifications with a matching date of birth will be excluded from receiving an invitation. After data validation, an SMS will be sent to the mobile phone number of the cases. The SMS will contain a link to the information sheet and consent form, securely stored on a RedCap database housed on the CALHN server.SA Health notification data does not specify the relationship between the contact mobile number entered in the online reporting system, to the case. However, there will be wording that ensures it is clear that a parent or guardian is required to consent to the study. This recruitment method will replace invites from phase 1 (WCHN) once governance approval is provided to avoid duplication of invites.

d. Consent

During phase 1 of recruitment a waiver of consent will be used to gather and report basic baseline clinical information of all COVID-19 and influenza cases that were swabbed at a WCHN site A waiver is already in place for all WCHN COVID-19 and influenza cases through the PAEDS study.

The participant information sheet and electronic consent will be accessible on the RedCap survey. The online consent will require an online signature of the parent and an online assent signature of their 12-18-year-old if they are also filling out the survey. A paper consent will be able to be provided upon request, but it will be the exception.

7. Participant Safety and Withdrawal

a. Risk Management and Safety

Whilst there are fewer risks than in interventional studies, some risks are described below with the mitigation strategies the study will use.

1) Psychological or Emotional Impact: The wellbeing questions may prompt emotions among participants and parents. The questionnaires are validated for children, adolescents, and their parents. Measures have been taken to minimise the risk of distress. Refer to Appendix A ‘Distress Protocol’.

2) Privacy and Confidentiality: The data will be collected using a password-protected RedCap database stored on an SA Health server behind an SA Health firewall. The database will only be able to be accessed by designated study staff, who are employees of SA Health. Identifiable data obtained from WCHN eligible cases will not be provided to non-WCH members of the study team without the participant's consent. Data will only be able to be extracted by the study PI, and no identifiable data will be stored outside of the password-protected RedCap database. The final dataset will be de-identified before being stored securely on a SA Health computer, only accessible to study staff. Information published from this study will not identify any participants involved in this study.

3) Economic Implications: It is expected that the survey will take approximately 10-15 minutes to complete. Efforts have been made to ensure that only necessary information is collected. Despite only having a small time requirement, it is still a burden in what can be busy family schedules. We have tried to ensure that the online platform remains flexible and that participants are compensated for their participation at $10 for each completed survey ($40 in total).

4) Coercion: The information sheet and consent form will clarify that participation is strictly voluntary and not linked to institutional benefits or repercussions. The reimbursement is modest, ensuring that the reimbursement is not an enticement.

5) Emotional distress from unwanted contact: There is a small risk that our SMS invite inadvertently reaches individuals who have passed away or do not wish to be contacted, potentially causing distress or privacy concerns. For phase 1 recruitment, EMR alerts will be checked prior to sending SMS invites. For phase 2, SA Health routinely check Births, Deaths and Marriages data each month to update their contact list, reducing the risk of causing emotional distress. It is a method they routinely use for all SMS contact with the public. To account for potential delays in reporting a child's death to the Births, Deaths, and Marriages register, a list of children and adolescents (0–18 years) who received care at WCHN and passed away in the previous month will be cross-matched with influenza and COVID-19 notifications. The PI collates a list of dates of birth for patients receiving palliative care or who have passed away. CDCB study staff cross-check this list against Influenza and COVID-19 notifications, and any matches by date of birth are excluded from receiving an SMS invitation. To minimise the risk of error, two CDCB study staff members will independently verify the age range and dates of birth identified for exclusion. SMS invitations are sent by CDCB using a secure, internally developed and managed SMS platform.

6) Possibility of failing to identify and manage untreated or inadequately addressed post-infectious symptoms. Refer to Appendix B: Follow-up of ongoing symptoms process.

b. Handling of Withdrawals

Participants may withdraw from the project at any time and refuse to answer any survey questions. They can withdraw by contacting the study team via email or phone and indicate if they wish to withdraw all their data from the study, future contact, or just future surveys. The REDCap database will have a variable that allows the PI to add the type of withdrawal and stop future automatic surveys from being sent out. The REDCap survey will also have the option to withdraw from the study within the questionnaire.

c. Replacements

Participants will not be replaced if they withdraw.

d. Distress protocol

Refer to Appendix A

8. Statistical Methods

a. Sample Size Estimation & Justification

The study is primarily descriptive. At the Women's and Children's Hospital, in the last 12 months, October 2022 to October 2023, there have been 800 COVID-19 and 1,100 influenza-positive swabs.

SA Health notification of COVID-19 PCR tests in those under 18 has been 335 PCR positive in the last 3 months, with 600 notified by RAT. There will be variations in the numbers throughout the study due to changes in testing and new variants. One possible change will be that RAT results will no longer be collected. Estimated invites to be sent to SA Health notified cases are 1,340 from PCR results and 2,400 from RAT results. Year to date 9,343 influenza cases under the age of 18 have been reported (with 8,270 notified between April and October 2023)

Assuming 15% participation, based on response rates from similar studies, there would be approximately 1,222 COVID-19 cases, with 2,800 non-COVID-19 cases from SA Health notification over the 24 months of the study.

b. Power Calculations

If the long-COVID incidence is between 1% to 6%, a sample size of 1,222 COVID cases allows for the incidence to be estimated with a precision ranging from 0.62% to 1.47%, with 20% loss to follow-up over 12 months (with precision defined as the width of a 95% CI around the estimated incidence).

Table 2 Precision estimates based on either the proportion of long COVID in the population (between 1-6%) and the sample size (between 550-2000)

	Proportion with long COVID		
	**1%**	**3%**	**6%**
**Sample size**	**Margin of error for incidence estimate**		
550	0.83%	1.42%	1.98%
1,000	0.62%	1.06%	1.47%
2,000	0.43%	0.75%	1.04%

Assume 360,000 population at risk (children under 18 in South Australia)

c. Statistical Methods To Be Undertaken

Analyses will follow a pre-specified statistical analysis plan. The cumulative incidence of PCC following COVID-19 (primary objective) and PAS following influenza will be estimated at 3 months post-infection, with 95% confidence intervals. The proportion of individuals with ongoing symptoms will be reported descriptively (n, %) at 6 and 12 months. The relative risk of developing PCC following COVID-19 versus developing PAS following influenza (objective 2) will be estimated using a log-binomial model. A multivariable logistic model will be developed for predicting PCC at 3 months (objective 3), incorporating pre-specified predictors such as age (treated as continuous), sex, pre-existing conditions, and socioeconomic status. Model performance will be evaluated using the area under the receiver operating characteristic curve, with internal validation by bootstrap resampling in line with TRIPOD recommendations.[1] The relatively small expected number of PCC events may limit the number of predictors that can be incorporated into the final model and hence its predictive accuracy. Linear and negative binomial mixed-effects models will assess differences between comparator groups over time in continuous (PedsQL quality of life and fatigue scores, objectives 4 and 5) and count (days absent, objective 6) outcomes, respectively. Log binomial models will compare the risk of developing PAS according to influenza vaccine uptake (objective 7) and the risk of PCC by COVID-19 reinfection (objective 8). The duration and clinical features of PCC and PAS will be described using summary statistics, with summaries at 3, 6 and 12 months. Descriptive analyses will be based on complete case data.

Handling of follow-up and missing data.

Outcomes will be summarised at the planned 3, 6 and 12-month assessments. Participants contribute data to each analysis for which follow-up information is available. For the primary objective, participants with PCC at 3 months who discontinue or miss assessments at 6 or 12 months will be treated as missing for whether symptoms were ongoing at these time-points. Multiple imputation may be considered for the prediction model, depending on the amount and reasons for missing data at 3 months when determining PCC.

9. Storage of Blood and Tissue Samples

N/A

10. DATA SECURITY & HANDLING

a. DETAILS OF WHERE RECORDS WILL BE KEPT & HOW LONG THEY WILL BE STORED

Data, including consent forms, annual progress reports, recruitment records and study notes, will be kept for 30 years after the research project completion. At completion of the analysis, a de-identified dataset will be stored on the SA Health server, accessible by the PI and SA Health delegate. This de-identified individual patient data (IPD) will also be made available on a case-by-case basis at the discretion of the study team and WCHN HREC. IPD data will only be available to achieve the aims in the approved proposal.

A separate list of participant's study ID, first names, last names, and dates of birth will be encrypted, password-locked and saved in a Vaccinology and Immunology Research Trials Unit (VIRTU) folder on the SA Health server. Accessible by the PI and SA Health delegate. The original database with identifiable data will be encrypted, password locked, stored on a SA Health server, and deleted from the REDCap after the analysis.

b. CONFIDENTIALITY AND SECURITY

In phase 1 of the study, basic baseline information of all COVID-19 and influenza cases presenting to a WCHN site will be collected by a WCHN employed level 2 Registered Nurse from EMR to provide baseline denominator data for those not enrolled in the study. Any identifiable information obtained from WCHN eligible cases will not be provided to non-WCH members of the study team without the participant's consent.

In phase 2 of the study, SA Health notifications will be used to identify potential participants. The SA Health Centre for Disease Control Branch (CDCB) utilises an existing SMS platform to manage the sending of invitations and provide access to the information sheet and consent form. Only aggregated non-identifiable data will be provided by CDCB to the study team for those who do not provide consent, as per Figure 3. Identifiable data from individuals contacted based on SA Health notifications will be provided to the study team only after they have given informed consent.

RedCap sits within the Central Adelaide Health Network Digital Health World and cannot be accessed outside the SA Health network. It is encrypted and password-protected.

For those that consent, the baseline, 3, 6, and 12 months survey data will be collected using a secure RedCap database stored on a SA Health server (CALHN), behind a SA Health firewall. The database will only be able to be accessed by designated study staff, who are employees of SA Health. Data will only be able to be extracted by the study PI, and no identifiable data will be stored outside of the password-protected RedCap database. The final dataset will be de-identified before being stored securely on a SA Health computer, only accessible to study staff. Information published from this study will not identify any participants involved in this study.

c. ANCILLARY DATA

N/A

Appendix A Distress Protocol

Introduction

This distress protocol is designed to ensure the safety and well-being of participants in our study, which includes sensitive questions on quality of life. Although such protocols are common in qualitative research, our study's inclusion of physical and mental health topics, school or work absenteeism, healthcare visits, and detailed quality of life assessments from the PedsQL questionnaires presents a minor risk of emotional distress to participants and their parents.

Managing Distress and Providing Support

To provide for a child and young person's safety, emotional and psychological security, and well-being, our approach includes:

- **Participant Information:** An information sheet that outlines the study's aims, potential emotional risks, and participant's rights.
- **Support Resources:** At the end of each survey, participants have information to access to a variety of support options:
    1. Contacts for 24/7 Mental Health Services (e.g., Beyond Blue, Kids Helpline for ages 5-25) for access to immediate counselling.
    2. Contact information for the 24/7 Healthdirect on-call service for after-hours health advice.
    3. A recommendation to see their regular GP for non-urgent health concerns.
    4. An email contact for a Registered Nurse from the study team for any study-related questions or issues.
- **Follow-Up Procedures:** Participants with quality of life scores below established thresholds, who have not previously sought healthcare advice, will be considered for telehealth contact (Appendix B). This step ensures they have the necessary support and offers additional help based on their personal needs and choices.
- **Feedback Mechanism:** At the end of each survey, participants will be given a free text option to give feedback on the survey process, including anything they found challenging. This feedback will enable continuous improvement of our survey questions and distress protocol.
- **Participant Autonomy:**
    1. At the start of each survey, participants are informed that they can skip any question they prefer not to answer.
    2. Participants are reminded that they retain the right to withdraw from the study at any stage, as outlined in the information sheet and within the survey instructions.
- **Experience of Researchers:**

  An experienced level 2 registered nurse with over 20 years of clinical and 10 years of research experience will handle the participants' queries and review the surveys. When required, advice will be sought from study team members, including paediatricians and child psychiatrist.

APPENDIX B FOLLOW-UP OF ONGOING SYMPTOMS PROCESS

Introduction

After participants complete the survey, a registered nurse or Paediatrician will review the responses to ensure that individuals with ongoing symptoms from COVID-19 or influenza are identified for further medical evaluation and care. This review assesses participants' health status and engagement with healthcare services to determine if a referral to a healthcare provider is necessary.

Telehealth Review Process

<u>Identification of Participants for Telehealth Contact with Severe Symptoms:</u>

Participants describing severe ongoing symptoms identified through the survey and not yet assessed by a medical professional (asked in the survey) will be contacted for a telehealth review. The following symptoms, among others, will prompt a follow-up:

- Constant headaches
- Difficulty breathing
- Chest pain
- Palpitations, high pulse, or irregular heartbeat
- Constant dizziness
- Problems with swallowing or chewing
- Seizures or fits
- Impaired movement control
- Speech and language difficulties
- Vision problems or blurred vision
- New allergies

<u>Identification of Participants for Telehealth Contact Based on PedsQL Scores:</u>

Participants with PedsQL scores below established clinically meaningful cutoffs[43] will be considered for telehealth contact if they have not previously sought healthcare consultation for their symptoms or reported a pre-existing condition. The cutoff scores are:

Child Self-Assessment

- Physical Functioning: 73
- Emotional Functioning: 60
- Social Functioning: 67
- School Functioning: 63

Parent Assessment

- Physical Functioning: 63
- Emotional Functioning: 63
- Social Functioning: 62
- School Functioning: 57

References

1. Moons KG, Altman DG, Reitsma JB, et al. Transparent Reporting of a multivariable prediction model for Individual Prognosis or Diagnosis (TRIPOD): explanation and elaboration. *Ann Intern Med* 2015; **162**(1): W1-73.
2. WHO Coronavirus (COVID-19) Dashboard. 2023. https://covid19.who.int/.
3. Hirschenberger M, Hunszinger V, Sparrer KMJ. Implications of Innate Immunity in Post-Acute Sequelae of Non-Persistent Viral Infections. *Cells* 2021; **10**(8).
4. Zimmermann P, Pittet LF, Curtis N. How Common is Long COVID in Children and Adolescents? *The Pediatric Infectious Disease Journal* 2021; **40**(12).
5. Pinto Pereira SM, Nugawela MD, McOwat K, et al. Symptom Profiles of Children and Young People 12 Months after SARS-CoV-2 Testing: A National Matched Cohort Study (The CLoCk Study). *Children (Basel)* 2023; **10**(7).
6. Vivaldi G, Pfeffer PE, Talaei M, Basera TJ, Shaheen SO, Martineau AR. Long-term symptom profiles after COVID-19 vs other acute respiratory infections: an analysis of data from the COVIDENCE UK study. *eClinicalMedicine* 2023.
7. Williams P, Koirala A, Saravanos GL, et al. COVID-19 in New South Wales children during 2021: severity and clinical spectrum. *The Medical journal of Australia* 2022; **217**(6): 303-10.
8. The impact of a new disease: COVID-19 from 2020, 2021 and into 2022: Australian Institute of Health and Welfare, 2022.
9. Davogustto GE, Clark DE, Hardison E, et al. Characteristics Associated With Multisystem Inflammatory Syndrome Among Adults With SARS-CoV-2 Infection. *JAMA Netw Open* 2021; **4**(5): e2110323.
10. Paediatric Inflammatory Multisystem Syndrome (PIMS-TS) in Australia. 2023. https://paeds.org.au/pims-ts/paeds-pims-ts-case-data.
11. Whittaker EA. Commentary: Post-COVID Condition in Children and Young People: Where Are We Now? *The Pediatric Infectious Disease Journal* 9900.
12. Bonilla H, Tian L, Marconi VC, et al. Low-dose naltrexone use for the management of post-acute sequelae of COVID-19. *Int Immunopharmacol* 2023; **124**(Pt B): 110966.
13. O'Kelly B, Vidal L, McHugh T, Woo J, Avramovic G, Lambert JS. Safety and efficacy of low dose naltrexone in a long covid cohort; an interventional pre-post study. *Brain Behav Immun Health* 2022; **24**: 100485.
14. Li Z, You Y, Griffin N, Feng J, Shan F. Low-dose naltrexone (LDN): A promising treatment in immune-related diseases and cancer therapy. *Int Immunopharmacol* 2018; **61**: 178-84.
15. A clinical case definition for post Covid-19 condition in children and adolescents by expert consensus. https://www.who.int/publications/i/item/WHO-2019-nCoV-Post-COVID-19-condition-CA-Clinical-case-definition-2023-1.
16. Sellers SA, Hagan RS, Hayden FG, Fischer WA, 2nd. The hidden burden of influenza: A review of the extra-pulmonary complications of influenza infection. *Influenza Other Respir Viruses* 2017; **11**(5): 372-93.
17. Brown M, Gerrard J, McKinlay L, Marquess J, Sparrow T, Andrews R. Ongoing symptoms and functional impairment 12 weeks after testing positive for SARS-CoV-2 or influenza in Australia: an observational cohort study. *BMJ Public Health* 2023; **1**(1): e000060.
18. Taquet M, Geddes JR, Husain M, Luciano S, Harrison PJ. 6-month neurological and psychiatric outcomes in 236 379 survivors of COVID-19: a retrospective cohort study using electronic health records. *Lancet Psychiatry* 2021; **8**(5): 416-27.
19. Al-Aly Z, Xie Y, Bowe B. High-dimensional characterization of post-acute sequelae of COVID-19. *Nature* 2021; **594**(7862): 259-64.
20. Jiang L, Li X, Nie J, Tang K, Bhutta ZA. A Systematic Review of Persistent Clinical Features After SARS-CoV-2 in the Pediatric Population. *Pediatrics* 2023; **152**(2).
21. Lopez-Leon S, Wegman-Ostrosky T, Ayuzo Del Valle NC, et al. Long-COVID in children and adolescents: a systematic review and meta-analyses. *Sci Rep* 2022; **12**(1): 9950.
22. Pellegrino R, Chiappini E, Licari A, Galli L, Marseglia GL. Prevalence and clinical presentation of long COVID in children: a systematic review. *Eur J Pediatr* 2022; **181**(12): 3995-4009.
23. Zheng YB, Zeng N, Yuan K, et al. Prevalence and risk factor for long COVID in children and adolescents: A meta-analysis and systematic review. *J Infect Public Health* 2023; **16**(5): 660-72.
24. Bosworth M, Shenhuy B, Walker A, et al. Risk of new-onset Long Covid following reinfection with SARS-CoV-2: community-based cohort study. *Open Forum Infectious Diseases* 2023.
25. Say D, Crawford N, McNab S, Wurzel D, Steer A, Tosif S. Post-acute COVID-19 outcomes in children with mild and asymptomatic disease. *Lancet Child Adolesc Health* 2021; **5**(6): e22-e3.

26. Britton PN, Burrell R, Chapman E, et al. Post COVID-19 conditions in Children and Adolescents at 3 months following a Delta outbreak in Australia: a cohort study. *medRxiv* 2023: 2023.03.14.23287239.
27. Coronavirus (COVID-19) at a glance – 9 September 2022. 2022. https://www.health.gov.au/resources/publications/coronavirus-covid-19-at-a-glance-9-september-2022 (accessed 11th November 2022).
28. Marra AR, Kobayashi T, Callado GY, et al. The effectiveness of COVID-19 vaccine in the prevention of post-COVID conditions: a systematic literature review and meta-analysis of the latest research. *Antimicrobial Stewardship & Healthcare Epidemiology* 2023; **3**(1): e168.
29. Razzaghi H, Forrest CB, Hirabayashi K, et al. Vaccine Effectiveness Against Long COVID in Children: A Report from the RECOVER EHR Cohort. *medRxiv* 2023.
30. Bonilla H, Peluso MJ, Rodgers K, et al. Therapeutic trials for long COVID-19: A call to action from the interventions taskforce of the RECOVER initiative. *Front Immunol* 2023; **14**: 1129459.
31. Emerging Priorities and Consumer-Driven Research initiative. Post-Acute Sequelae of COVID-19 Research Plan: Australian Government Department of Health and Aged Care, 2023.
32. Carson G, Long Covid Forum G. Research priorities for Long Covid: refined through an international multi-stakeholder forum. *BMC Med* 2021; **19**(1): 84.
33. Sachs JD, Karim SSA, Aknin L, et al. The Lancet Commission on lessons for the future from the COVID-19 pandemic. *Lancet* 2022; **400**(10359): 1224-80.
34. Munblit D, Sigfrid L, Warner JO. Setting Priorities to Address Research Gaps in Long-term COVID-19 Outcomes in Children. *JAMA Pediatr* 2021; **175**(11): 1095-6.
35. Abbasi J. The US Now Has a Research Plan for Long COVID-Is It Enough? *JAMA* 2022; **328**(9): 812-4.
36. Long COVID sufferers face six months wait for SA help. https://indaily.com.au/news/2023/10/19/long-covid-sufferers-face-up-to-10-months-wait-for-sa-help/?utm_medium=email&utm_campaign=InDaily%20Lunchtime%20%2019%20October%202023&utm_content=InDaily%20Lunchtime%20%2019%20October%202023+CID_89ccb5bf6e7846dac3ab22b46e0b5586&utm_source=EDM&utm_term=Long%20COVID%20sufferers%20face%20up%20to%2010%20months%20wait%20for%20SA%20help.
37. Paediatric Active Enhanced Disease Surveillance (PAEDS) 2022. https://www.ncirs.org.au/our-work/paediatric-active-enhanced-disease-surveillance-paeds (accessed 11th November 2022).
38. Australia Community Profile - South Australia Language used at home. 2021. https://profile.id.com.au/australia/language?WebID=130&EndYear=2001&DataType=UR.
39. International Severe Acute Respiratory and Emerging Infection Consortium (ISARIC) - COVID19 Clinical research resources. https://isaric.org/research/covid-19-clinical-research-resources/2023).
40. Spahic JM, Hamrefors V, Johansson M, et al. Malmo POTS symptom score: Assessing symptom burden in postural orthostatic tachycardia syndrome. *J Intern Med* 2023; **293**(1): 91-9.
41. Varni JW, Seid M, Kurtin PS. PedsQL 4.0: reliability and validity of the Pediatric Quality of Life Inventory version 4.0 generic core scales in healthy and patient populations. *Medical care* 2001; **39**(8): 800-12.
42. Varni JW, Burwinkle TM, Szer IS. The PedsQL Multidimensional Fatigue Scale in pediatric rheumatology: reliability and validity. *J Rheumatol* 2004; **31**(12): 2494-500.
43. Huang IC, Thompson LA, Chi YY, et al. The linkage between pediatric quality of life and health conditions: establishing clinically meaningful cutoff scores for the PedsQL. *Value Health* 2009; **12**(5): 773-81.
